# Unexpected tissue and the biobank that closed: an exploration of value and the momentariness of bio-objectification processes

**DOI:** 10.1186/s40504-015-0032-0

**Published:** 2015-12-02

**Authors:** Neil Stephens, Rebecca Dimond

**Affiliations:** Department of Social Sciences, Media and Communications, Brunel University London, Kingston Lane, Uxbridge, Middlesex UB8 3PH UK; School of Social Sciences, Cardiff University, 12 Museum Place, Cardiff, CF10 3BG UK

**Keywords:** Biobanking, Bio-objectification, Bio-objects, Value, Waste, Closure, Momentariness

## Abstract

Unanticipated situations can arise in biobanking. This paper empirically documents unexpected situations at the anonymous biobank ‘Xbank’. Firstly, Xbank received an unexpected and significant quantity of tissue from the historical archive of a hospital network. Secondly, Xbank had its funding withdrawn before the designated end date for the grant, meaning the bank needed to either re-house or destroy its holdings. This paper articulates and uses the theoretical frameworks of bio-objectification and tissue economies to analyse the experiences of Xbank and draw out further implications of the potential precariousness of biobanking practice. The case study allows an inspection of how the value of tissue is configured and reconfigured as institutional contexts shift. We introduce the notion of momentariness as a way of grappling with the related temporariness and perpetualness of biobanking practice in both a theoretical and practical policy context.

This paper considers critical issues on the production of value in the increasingly prevalent Biobanking sector. In particular, it focuses upon an anonymous disease specific biobank pseudonymised here as ‘Xbank’. Over the course of 6 years Xbank was initiated, established, developed and then subsequently shut down due to broader funding decisions. The paper documents a specific episode in Xbank’s activities, where a historical archive of unwanted tissue entered Xbank’s biobanking infrastructure to be catalogued and prepared for distribution for research purposes. However, during this period Xbank staff received notification of the banks imminent closure. Subsequently the paper details a period when the tissue from the diagnostic archive shifted from a position of valueless to valued, then to a new state of precarious valueness and then reconfigured into new forms of value. We use this to explore the momentariness of bio-objectification. In summary, our core theoretical argument is: (i) biobanking activities, both anticipated and unanticipated, shape that status of tissues and how they are valued, (ii) the relationship between the socio-technical context of biobanks and the tissue within them is productively characterised with the notion of momentariness, that captures both temporariness and perpetualness of status and value, and (iii) combining the theoretical frameworks of (a) bio-objects and (b) tissue economies provides a robust platform for analysing these issues. Our Xbank case study illustrates the precariousness of the value of tissue, and the precariousness of biobanking institutions, as unanticipated events hasten reconfigurations of the status of both.

From a policy perspective, issues of the reconfiguration of the status and value of tissue demand increased attention in the light of work by Zawati et al. (2011) and Cadigan et al. (2013, 2014) highlighting the under reported and under analysed issues associated with biobank closure. By focusing on these practical issues, our paper makes a contribution to the emergent ‘Sociology of Biobanking’ (Lipworth et al. 2011). However, unlike Lipworth et al’s work, which focuses upon patient donor experiences and expectations of donating material to tissue biobanks, our project explores the internal workings of the biobanking infrastructure as a socio-technical accomplishment, aligning disparate objects and actors through a process of bio-objectification.

## Theoretical approach: bio-objects and the tissue economy

The paper draws upon (i) the bio-objects theoretical framework (Vermeulen et al. 2012; Gajović 2014) and (ii) Waldby and Mitchell's (2006) tissue economy approach. In doing so it demonstrates the compatibility of these approaches through the careful inspection of a specific empirical case study.

The bio-objects framework focuses upon socio-technical phenomena that can contest and reconfigure the notion of life (Webster 2012). Typically this is imagined as a biological material cast within specific institutional relationships. Core to this perspective is a recognition that the status of these material phenomena is most usefully understood as a process – the process of bio-objectification – as opposed to a steady state, as shifts in socio-technical-material relationships change the phenomenon’s status. The empirical work presented here provides a clear example of the relationship between status and context. As Holmberg, Schwennesen, & Webster argue:

“bio-objectification processes are not linear or have a specific path-dependency. Bio-objectification can start at one point, go through institutional transformations, come to a halt or be silenced, and then revitalized at a later point. This means that bio-objectification explicitly includes consideration of organizational and institutional processes and the ways in which the governance of bio-objects can bring closure and stability to them, but which is always likely to leave open the possibility of new contestation and debate in the future” (Holmberg et al. 2014 p12).

Extending this, it is important we recognise that these “generative relations”, meaning the connectivities between biological material and the broader economic, social or political contexts, are co-produced with the biological phenomenon through a process of bio-objectification (Tamminen and Vermeulen 2012). Indeed, as Eriksson (2012) argues in the case of the pluripotency of human embryonic stem cells, processes of bio-identification and the generative relations they co-produce are as much premised upon what the bio-object could become (and the institutional processes that will afford that) as the status of the bio-object today. In this paper we capture and augment this perspective through elaborating the notion of momentariness.

We take our lead from the bio-objects tool-kit developed by Webster (unpublished), a framework providing concepts suitable for application by anyone interested in the revealing capacity of the bio-objects approach. Summarising his own argument, Webster states:

“The term “bio-object”… refers to a socially potent biotechnological entity which generates controversy due to its potential challenging of established classifications. The concept thus entails four different dimensions:

1. Matter out of place and the process of bio-objectification: Challenging classifications
2. Controversy in one or more arenas of society: Challenging social order
3. Organizational and institutional processes: Technologies and labour involved, that are sometimes themselves problematized: Challenging ‘discovery’
4. Bio-identification: Consequences on individual, symbolic and structural levels; changing social relations, the emergence of new categories and identities: Challenging social relations” (adapted from Webster, unpublished, p2).

The first dimension – matter out of place – clearly invokes Douglas' (1966) account of how people classify things. Bio-objects cross boundaries, or hover on boundaries as boundary crawlers (Chrupek et al. 2012), shifting in and out of the classifications we use to order experience. It is through these shifts between classifications that they become entangled with controversy, as in the human embryo that becomes a stem cell line, or move between statuses of value, as is the case in our empirical study of biobanking. The bio-objects theorist must map these boundary shifts and the socio-technical relationships co-produced. Controversy is itself Webster’s second dimension, and his toolkit urges the analyst to document how controversies are formed and contested, made robust or silenced. The third dimension reiterates the earlier point of the centrality of institutional processes and their co-production with bio-objects. But perhaps most importantly, Webster’s account introduced the fourth dimension, the notion of bio-identification, to the theoretical framework. The term refers to moments when the identity of a biological phenomenon is stabilised. As our empirical work will show, it is key to retain this notion of bio-identification as momentary (sometimes prolonged) as opposed to a step change in status because bio-identification can, and sometimes does, become undone as the socio-technical arrangements with which they are co-produced shift.

Within this broader Bio-objects framework we also embed the work of Waldby and Mitchell (2006) and their exploration of the tissue economy. The tissue economy is “a system for maximising [tissue’s] productivity, through strategies of circulation, leverage, diversification, and recuperation” (p31). They develop Callon (1998) to articulate the work of biobanks and other actors within the tissue economy to entangle, disentangle and re-entangle tissue within different socio-cultural contexts and mechanisms of exchange, using the passage of human embryonic stem cell lines into the UK Stem Cell Bank as an example.

Elsewhere we have explored these entanglements and re-entanglements based upon empirical evidence collected at the UK Stem Cell Bank (Stephens et al. 2008). In this paper we extend this analysis by bringing empirical scrutiny to Waldby and Mitchell’s conceptualisation of waste, as embedded within the bio-objects framework, through an inspection of the multiple entanglements of tissue as waste and value at Xbank.

Waldby and Mitchell argue that waste is present in any economy. The construction of particular material as waste is always related to its position within a constellation of values and network of exchange. Showing clear synergy with Webster’s (unpublished) account of Douglas (1966), waste is understood as matter out of place. For Waldby and Mitchell, waste is always circulated from being unprofitable objects into potentially profitable contexts. Their examples include babies’ foreskins, cord blood, and cancer tissue as instances of waste becoming valuable. Waldby and Mitchell use the term speculative biology to capture the manner through which waste produces value via the capacity of tissues to open the possibility for new and unexpected forms of value. Biobanks like Xbank exist to maximise the opportunity of this speculative biology by cataloguing and making visible otherwise locally collected and invisible tissue to broader demand from research professionals. In doing so they are key institutions in bio-objectification processes.

Another important element of Waldby and Mitchell’s framework is a recognition that human tissue is frequently overdetermined in that tissues can be simultaneously subject to multiple, often competing, regimes of value. Each competing regime articulates a different configuration of the tissue’s meaning and most pertinent features. Moments of contestment between multiple regimes of value can provoke controversies that challenge the co-production of a tissue’s value and the socio-technical arrangements that support it. The movement of tissue across both physical and symbolic locations involves “hierarchizing the values associated with tissue productivity” (Waldby and Mitchell 2006 p31), bringing further tension between competing regimes. Later we expand upon this with the example of competing value regimes placed upon a patient’s tissue by the patient, by a hospital holding the tissue, by Xbank itself, and by potential researchers. The case study will also make explicit the relatedness of the regimes of value of the bio and the non-bio through making clear the role of material things – paperwork, boxes, and buildings – in processes of bio-objectification.

This paper works to show the compatibility of the bio-objects and the tissue economy approach through their synergetic application to our Xbank empirical case study. We also augment these ideas by highlighting the momentariness of bio-objectification. Importantly, we retain the dual meaning of momentary as both of lasting only a moment, and to be occurring at every moment as a form of the perpetual. In doing so we highlight both the contingency and continuity of bio-identification within the tissue economy.

## Methods

The paper is based upon data collected through 14 interviews with six individuals conducted over a three year period, as well as observational research conducted at Xbank organised events. Since Xbank only had four members of staff for most of the data collection period this represents a detailed dataset. The individuals quoted remain anonymous, as does the identity of Xbank, all institutions it has worked with, the exact times and dates of events described, and the specific disease focus it operated within. This level of anonymity is a requirement of the access that has been negotiated to the site. Occasionally interview quotations have been edited to assure this anonymity. The interviews were conducted by Stephens and subsequently coded by Dimond and then analysed and written up by both. Key to our message is the situated labour of biobanking work in bio-identification, so we use detailed interview extracts to evidence participant accounts.

## Bio-identification at a functioning biobank: taking in tissue at XBank

Xbank was funded by three charitable and governmental organisations. It was intended to address bottlenecks within the national movement of quality assured human tissue towards researchers working in a disease specific context. New emphasis on tissue flows following increased attention on ensuring their correct ethical guardianship had mounted in the preceding decade leading to restricted exchange under new regulatory scrutiny and bureaucratic accountability. Xbank was intended to address this by forming networks with hospitals and embedding staff within them to collate tissue and blood samples that had been deemed as ethically sourced to be logged within their computer systems and centrally stored in Xbank’s biobanking facility. Xbank were also charged by their funders with developing best standards within their disease specific field of biobanking and lead on sharing those standards via a newly established disease specific biobanking network. While continuing to engage in working with hospitals, Xbank were contacted by a regional hospital group based in a large city, here pseudonymized as ‘Ytown’, about a significant quantity of human tissue taken for diagnosis that they had collected over the last 100 years that was stored in a building that was due to be demolished:

Senior Manager: “One significant collection we have is this diagnostic archive from the entire hospital collection from Ytown from about the turn of the last century up to about 1978. Now it is uncatalogued, it’s massive, it’s very diverse and it was destined for destruction… we kind of rescued it from the fire so to speak. In the belief that someone would be able to get some use from it”.

This was tissue that had had ontological significance at the period of collection. Between 1900 and 1978 when this material was removed from individuals undergoing treatment it had a stabilised bio-identity as diagnostic material deemed valuable to understand the individual’s health condition and potentially that of family members, and was typically valued as such by both patient-families and hospitals. However over time, for the hospital, this material became clinical waste and matter out of place (or matter without a place as the building was due for demolition), losing some of its ontological significance and value. This given, it did not fully become ontological waste for the hospital. While the tissue did lose its use value it remained worthy of regard and recognised as potentially valuable in other contexts as evident in the hospital’s efforts to circulate it further through the tissue economy. We do not know what difference the passage of time had on the ontological status of this tissue in the patient-families regimes of value, but it is clear that by this point the tissue’s entanglement in the economic-property relations of the land, the building, and the hospital afforded priority to the value regimes of the hospital. The tissue had outlasted the organisational and institutional processes core to its bio-identification as the co-produced productivity of the tissue and the building that housed it declined relative to the reuse value of the land. The prolonged momentariness of valued tissue and valued tissue storage facility came to an end.

The relationship between the value of the tissue, the value of the building storing it, and the value of the land it was built upon, evidences the connectedness of the value of the bio and the non-bio. It also highlights the presence of multiple regimes of value as the tissue was ascribed worth by both patient and hospital. The work of Xbank substantiates a third regime of value, as Xbank conducted the speculative biology of attempting to re-entangle this tissue as exchangeable and valuable for research. As we shall see, this endeavour would require significant labour, networking, and bureaucratic and promissory work.

In order to be suitable for exchange within the tissue economy, this material would need to be logged within standardised forms of bureaucratic accountability that placed the material within visible sets of order (cf Eriksson and Webster 2015). This would prove quite a challenge considering the quantity and condition of the material as it entered Xbank:

Manager 1: “We’ve got materials from this diagnostic archive, 300,000 specimens. A roomful of paperwork and three 18 tonne lorries carried all the specimens and paperwork from Ytown and offloaded it, the paperwork came here because it’s got patient identifiable information so that’s within our control, the samples are all at [a hired sample storage space]. It was just unloaded from the lorries into their workshop, and it’s unsorted, it came from a number of different hospitals in Ytown. I got a spreadsheet saying, blue label with pink stripes is from this place, and all needed sorting out”.

However, just as the tissue had arrived, staff at Xbank were informed that their funders had decided they would not continue funding Xbank beyond its initial five year grant. The funders felt Xbank had not collected enough tissue through the anticipated route of patient donations freshly collected via their hospital networks in the time allocated. Initial plans written during negotiation with each hospital had suggested a certain level of tissue flow into Xbank. However practical collection issues within the hospitals had led to significant under performance. Essentially Xbank was not collecting enough tissue at high enough speed and the funders decided that, in a context where other biobanks in the disease area existed within the country, there was no need to continue funding Xbank’s active biobanking work (cf Stephens and Dimond 2015 for an extended analysis of Xbank’s closure).

Despite this blow, the funders did not decide to shut down Xbank altogether. Instead they suggested that what had been Xbank’s secondary function, to develop best standards and form a UK wide disease specific biobanking network, was proving successful, and Xbank’s remit should be reconfigured so that this became their primary and only focus. Developing best practise is significantly cheaper work than active biobanking. After redundancies and significantly decreased spend on the infrastructure required to handle tissue in controlled conditions the remaining budget could cover the costs for Xbank to continue to function in this new role for around two more years.

This was a significant moment of institutional reconfiguration for Xbank. The workforce was cut by more than half and their goals significantly reconfigured. As part of this the current collected tissue needed to be redistributed or destroyed. Most of the tissue collected from contemporary patient-donors undergoing treatment in hospitals was returned to where it was collected. However, the biggest quantity of tissue held by Xbank at this time was the Ytown diagnostic archive. Xbank would now have to formulate mechanisms for further circulating this material within the tissue economy.

## Seeking value after the cessation of biobanking activity

The significant quantities of material from the Ytown diagnostic archive held at Xbank still needed to be sorted. It now also needed to be moved on from Xbank. The institutional reconfiguration of Xbank had once again placed the bio-objectification of this tissue in jeopardy. Previously the credibility and presumed institutional stability of Xbank had positioned this tissue, through Xbank’s role as speculative biologists, as of value or as of potential value to someone else. The reformulation of their role by their funders meant Xbank could no longer act as an institutional location safeguarding and entangling this tissue within networks of stable supply. Instead, Xbank needed to find new mechanisms for transferring this waste into tissue commodities in new institutional contexts with new promissory narratives; otherwise it would be re-entangled within networks of waste. Essentially for Xbank this meant finding a new home for this tissue.

This was a challenge for Xbank to arouse interest in sufficient individuals and institutions who have the logistical capacity to cope with this quantity of material:

Senior Manager: “If all of a sudden we had lots of people knocking on our door saying ‘yeah we want those samples’ then it would be a major exercise to go extracting them from this massive resource. But I would be quite confident that if that were the case I would say to the funders ‘look people are interested in these’, then they’d be saying ‘okay well let’s hold onto them until we’ve served their interests’. It’s in the absence of interest that we have a deadline to dispose of them and at the moment we’ve got pretty limited interest, which in itself is interesting because of course we’re always being told there aren’t enough samples, or we’re being told access to samples is difficult and you know as you’ll see from that leaflet we’ve been pretty open about we’ve got them, ‘come and get them’ you know. If you’ve got an ethically sound use that isn’t going to cause great problems, they’re here for the having, just let us know and come and get them. People haven’t been beating down our doors”.

The interviews conducted at the time of the quotation above demonstrate a lack of confidence that up to that point research institutions genuinely had an interest in taking this material on. However, Xbank continued to present the tissue as valuable, hoping to inspire external bodies to take on their holding and provide the institutional and bureaucratic contexts that could make this claim to value more robust. In practice, this resulted in a targeted advertising and awareness raising campaign:

Manager 2: “[We wanted] to not pretend that they’re anything other than they are, which is a diagnostic archive, and they’re paraffin embedded samples and we can’t kind of guarantee the quality of them, but we do need to find an eye-catching way of attracting attention. So we developed a slide for use at conferences… an e-flyer that we sent out actually on Monday, to a list of people who attended our January meeting… used some of our contacts with some of the other clinical trials networks to distribute to their membership…”

Interviewer: “Did you consider other ways of trying to communicate?”

Manager 2: “We did and we considered whether or not to do anything with the media, a press release or something like that, and we just felt that actually the tone of that kind of a communication was very difficult to get right and that it was much better as a direct contact between us and potential researchers than make it look like… it’s a kind of garage sale… you just have to be quite careful about how we did it”.

This quotation demonstrates the perceived importance of nuanced representations of both Xbank as the provider of tissue and the context of the tissue itself. Judgements were being made about the appropriateness of particular mechanisms of awareness raising to specifically position the potential of disentangling and re-entangling this tissue in outside bodies’ own institutional arrangements.

Despite the initial scepticism noted above, within 5 months the awareness raising campaign had proved more successful than some members of Xbank’s staff feared it may be:

Manager 2: “The first thing probably was promoting the samples… And it’s interesting because we sent out in a few emails to our distribution list [and] a couple of other circulation lists. And then [one specific email list] and literally within hours of them sending it out, we got masses. We got loads of replies… something like fifty five enquiries… So we logged them all and we sent people information about the collection and how we would have to go about searching for their samples. We had a couple like saying ‘I’m interested in eyes. Do you have any eyes?’ [Laughing]. No we don’t because it wasn’t an ophthalmology centre and so things like that. So we checked with [the Senior Manager] are we likely to have X or Y? Some people we just sent an immediate ‘sorry we won’t be able to help’ and other people we said, ‘yes we probably will have those in the collection. It’s a paper search because they’re so old and this is the sort of timescale. If you’re interested in taking it further look at our access policy which was on the website, the pricing policy which was on the website, and fill-in the application form and then we’ll take it further but you have to formally apply and then we can take it further’. And out of those people I think fourteen completed a formal application. And so Manager 1 is at the moment going through trying to find those samples for people. So yes it was good. So we realised that people are interested in paraffin fixed sections”.

This quotation demonstrates the importance of locating tissue within specific social networks in the production of value and locating the tissue within specific interest cohorts of speculative biology. In the context of biobanking it is these mechanisms that are core to the co-production of generative relations and bio-identification. The awareness raising campaign initiated a process of ongoing negotiation and alignment between specific subsections of the larger tissue collection, with specific sets of interests and logistical capabilities within the broader networks of disease specific biobanking and research in the country. The importance of bio-objectification as a process based on momentariness is clear here as both the temporariness of status and value premised upon contingently configured socio-technical alignments, and the perpetuity of recycling value and waste articulated by Waldby and Mitchell (2006), is apparent. The role of timespans in momentariness is clear in this account from the senior manager:

Senior Manager: “In terms of the Ytown diagnostic archive set that we’ve got, then again, with Manager 2’s communication skills, I think we got more than 50, almost 60 expressions of interest, it turned into 14 proper applications. We facilitated 1500 cases going to [a new disease specific biobank]. And we’re trying at the moment to find appropriate cases to fulfil the requests of these 14 other investigators. As a consequence the archive itself effectively has had a stay of execution to the end of May. If there had been no interest we would have destroyed it by now. After which it probably will be destroyed and I think that’s a bit of a shame. But we can’t financially sustain it, it costs us about €2000 a month to keep this stuff. And without there really being a real steady trickle of interest in it, then I think its time is up”.

The success of this networking activity and the sharing of a promissory narrative around the value of this tissue was used by Xbank staff to convince their funders to continue funding Xbank’s holding of this material while the subsequent labour of cataloguing and entangling the material within systems of bureaucratic accountability commenced. Importantly these bureaucratic records are new records, the systems of documentation of the moment. The historical documentary records passed a different path:

Senior Manager: “I suppose what bothers me even more than the actual physical samples is we’ve also got all the paper records. And collectively, the paper pathology records and the samples together, represent the accumulated health history of the city of Ytown through all its various hospitals, over a period of about 100 years. And I’m almost gobsmacked that nobody is interested in that. We have offered the paper records to a national archive, they’re not interested. So the paper records that contain identifiable information will have to be destroyed”.

As we will see in the next section the tissue and the paperwork, quite literally made of paper, requires maintenance and ratification to ensure the ongoing entanglement of this once waste material in processes of bio-identification.

## Sorting the collection: value as bureaucratic accountability and exchange

The tissue now took on a form of value. It was wanted and new sets of expectations and promissory narratives were being formed around its use. Yet the tissue still needed to be collated in a form suitable for continued exchange within the tissue economy. Achieving this required the continued labour and skills of the Xbank staff to respond to the condition of material that had lost its ontological significance within the Ytown hospitals, and allowed to lose the work of maintenance and care that is required for bio-objectification in biobanking. The extent of this loss of care is evident in the following quotation:

Manager 1: “So I went to the storage site and made sure I could actually find the blocks that went with the reports. I haven’t done that exhaustively but I know where the blocks are and I can pull them out. But I found that the way the samples had been transported, they’d been transported in filing drawers. All the recent samples are in cassettes; little plastic, solid blocks that you place the tissue in, fill with wax, and then you get the three of them together. The wax with the sample and still in this plastic; very easy to file standing up. They’re all the same shape, they’re designed to fit one inside the other, so beautiful filing, all in order from about 76 onwards, which is only 2 or 3 years of our collection. Before that, it’s just the wax block and the labelling with the wax block is a bit of paper embedded in the wax. Now that ain’t easy to read, and a lot of those drawers appear to have been shaken around. I’d describe them as they look like someone’s taken the drawer out, tossed it in the air, and just caught the blocks wherever they could! You know, like tossing a pancake; they’re all over the place. It’s really difficult to find a specific block in that lot. And that’s part of the reason I went for the more up to date samples, because they’re in cassettes. They’re beautifully in order and it’s easy to pick them. Where I wasn’t able to find a number of samples in the past few years, I have on a couple of instances gone back through the drawers and just tried desperately to find them. It’s just not productive of time. You know, you can spend a couple of hours finding one or two samples in the drawer”.

The value relationship of bio and non-bio is evident in this account of the cassettes, the wax blocks, the labels and the draws, as different material forms afforded different levels of access to the tissue block sought. This ongoing ordering of tissue became a necessary pursuit for the staff of Xbank as they dealt with dusty cassettes and unordered wax blocks of tissues with differing levels of comprehendible labelling. In addition to the physical work of ordering the tissues, further expertise and labour was required, not just to find the material, but to find out what it was:

Manager 1: “Okay, practically in our situation, four of us working here; there’s only really [the Senior Manager] and I with any technical knowledge and she’s the one who’s the expert… so practically what I did was I produced a database of who wanted what; what criteria they had written down, what special requests they had – don’t give us any post mortem tissue or I need normal tissue linked to this [specific disease]… So I typed up what they’d written, took it to [the Senior Manager] and said I’m going to start looking for these. What other terms should I be looking for, what are the synonyms, and bounced it all off and got her advice, added all that to my database. And then had to decide where I was going to start looking for them… I’ve realised that [the Senior Manager] is going to have to sit down with blocks and slides and a microscope and try and identify which of them is exactly what we’re looking for to send off to the researcher”.

Ordering represents familiarity and expertise within systems of cataloguing. Such systematic positioning of this tissue is essential to its ongoing bio-objectification. Beyond cataloguing and identifying the tissue itself, the initial records which initially catalogued and identified the tissue needed to be catalogued and identified themselves. The condition of these documents, again, posed significant challenges for Xbank staff:

Manager 1: “What they tend to do is collect up the records over a year and then get them bound. So the binders themselves are falling apart and the spines are turning to dust as you touch them and you need a lab coat and gloves to go in the room, possibly a mask to keep the dust out of your lungs. But in terms of actually reading and extracting the data, it’s all there. There’s a few that have been in a basement in a mortuary and got wet, which are more difficult to deal with. And some of them are carbon copies; like literally old-style carbon copies so they’re all a bit fuzzy. But they are histology reports so the terminology is familiar and even where you can’t make out every letter in the word, you can understand what it is they’re talking about. And you can pick out what you want… And the numbering system that all the different hospitals have used for the blocks is the same – well, is often the same. So on the 1st of January they start at 1/78; 2/78, so if we’ve got samples from five or six hospitals, we’ll have five or six labelled 1/78. And it’s knowing which hospital they came from that enables you to find the right report to go with it”.

The passage of time had led to the loss of ontological significance of this tissue, and with it a progressive breakdown of the systems of maintenance that had retained the systems of bureaucratic accountability that made the tissue valuable. Xbank had to address this passage of time to the extent that in some instances they had to recreate the technological environment in which the tissue had initially been collected:

Manager 1: “There’s a local museum that the library put me in touch with, and I went and talked to them and they’re prepared to lend us their microfiche reader to bring back here. It will mean projecting the microfiche onto the screen and then taking a photograph of it, and then putting it back into the computer and using that to generate a report for the researcher. So again, it’s a complicated and time consuming way of doing it”.

The case of the microfiche reader demonstrates the socio-technical form that institutional processes take. The moments of bio-identification of the tissue as diagnostic brought with it certain constellations of specific and generic technologies that needed recreating in this work of re-entangling value. Once subsections of the tissue had been ordered and subsections of the paperwork had been ordered, the two needed to be positioned in alignment via an explicit mechanism for identifying which paper records related to which tissue samples.

At this point in our account the tissue from the Ytown diagnostic archive has gone from a position of value as something that directly benefited the health status of the patient to something that had lost its ontological significance over time. When initially donated to Xbank a new speculative biological value was associated with the material via Xbank’s own guardianship, entangling it within their own status as a stable institution and their promissory narrative of providing quality assured tissue for research. The subsequent cessation of biobanking activity at Xbank then undermined this newly created value by removing Xbank’s institutional stability that had lent support to the process of bio-objectification. The subsequent successful awareness raising campaign, the processes of negotiation aligning external institutions’ needs from particular subsections of the tissue collection, and the sorting and labelling labour engaged in by Xbank staff, brought new forms of value to the tissue held within their soon to be dismantled Biobanking infrastructure. However, to substantiate this value, the tissue would need to leave the newly precarious and transient space of Xbank’s Biobanking infrastructure and be passed on to new custodians.

## New sites of speculative biology: moving tissue out of Xbank

The majority of requests for material arising from the awareness raising campaign were for relatively small quantities of tissue from perhaps five cases to around 250 cases. By some degree the biggest accession of this material came from a single newly established biobank. This group recognised the potential of this tissue collection to form the initial basis of its ongoing donations of further material, which would provide a substantial basis for its holdings. However, taking such a quantity of material would also prove a significant activity on its own:

Manager 1: “Two guys from [the new biobank] came over; stayed for a week, went through the records, picked out the samples that they were interested in, and then went over to [the storage facility] and pulled blocks and couriered them all back to [the new biobank]. So I think it was something like 1500 cases they took away which felt good. This is what this material should be used for… Initially they wanted to take all of the [body part specific] material back to the 1800s and then they saw the size of the problem when they got here and two of them for a week, there’s no way they were going to find everything – particularly when we were going through drawers trying to pick out from amongst a mess of wax blocks. So what they decided to do was to take all the samples from the years that were in cassettes and then take samples at 10 year intervals going backwards, so that they could look at differences in diagnosis, tissue processing and storage, artefacts and survival of the sample. That’s good biobanking research, as much as looking at the disease itself”.

The labour conducted by the staff of the new biobank, both on site at Xbank and within their own biobanking infrastructure, lend further institutional credibility to the material, embedding it within a new promissory narrative associated with that institution, redefining it within their own bureaucratic system, and entangling it within a new form of value that could still subsequently be circulated through the tissue economy to new sets of researchers via their own biobanking practises. This is a new moment of bio-identification, with a new institutional process that puts matter in a new place with a new regime of value.

## Disposing of valued waste, and waste as value

While some of the Ytown archive had successfully been circulated into new networks ratifying its status, a significant proportion of it was not. It became, once again, within the economic context at that moment, recognised as waste. No longer able to retain this material within Xbank’s own Biobanking infrastructure, the material continued to circulate through the tissue economy into new spaces where waste could become value in different forms:

Manager 1: “For the data, interestingly the records are more of a problem than the samples themselves. Samples themselves are human tissue so you can go on the official website and look up the code of practice for the disposal of human tissue and you find out that your choices are incineration, burial or cremation. It would be incineration. We’re not going to bury three 18-tonne lorries’ worth of wax blocks. Then finding someone to do that at a cost we can afford isn’t as straightforward as it ought to be because there’s so much of it and generally hospitals don’t dispose of that huge amount at one go. So we’re still working on that one… Data is another issue. We’ve looked at what are our responsibilities with the data. And there are guidelines on storing this type of thing. But it doesn’t stop us destroying the data”.

The samples and the records once again became waste as the infrastructural supports of its bio-objectification ceased to be economically sustainable. However, importantly, this waste did not become so valueless that any mode of disposal was deemed appropriate. Instead, even as clinical waste, the ontological status of the tissue and the social relations co-produced with it demanded that even in destruction the tissue could not be left to be dirt: there remained an imperative for it to be matter in place, with that place being the documented and accredited spaces of tissue disposal. In this regard the tissue remained a form of valued waste, a matter afforded a specific form of care, even as it was destroyed. In practice this destruction came through incineration, and with it occurred the final moment of speculative biology documented here as the tissue provided a source of income for the tissue disposal companies who had licence to destroy clinical waste. In this final moment of circulation the tissue shifted one last time from a context of unprofitability into a context of profitable destruction.

## Conclusion

In this paper we have made clear the compatibility of the bio-objects and the tissue economy framework for analysing change in contemporary biomedicine. From the starting point of a shared focus on waste and matter out of place we have shown the two frameworks to support and confirm each other, for example as Waldby and Mitchell's (2006) account of speculative biology synergises with Eriksson's (2012) argument that processes of bio-objectification can be premised upon what a bio-object could become.

The paper has reported two unanticipated turns in our empirical case study of Xbank. The first is the unexpected availability of a significant historical archive of tissue. The second is the closure of the biobank. Both allow us a greater understanding of the precariousness, and the momentariness, of bio-objectification processes. This precariousness highlights the vulnerabilities of this co-productive process as reconfigurations anywhere within the constellations of bio, social, technical, and institutional can reverberate and impact elsewhere. We have made explicit the relatedness of bio and non-bio, and the role of multiple regimes of value in both supporting and contesting the status of tissue and the social-technical forms with which it is co-produced through generative relations. The contingency of momentariness is also clear, and we have empirically demonstrated the claim by Holmberg et al. (2014) that bio-objectification processes have “no specific path-dependency”, and that they “come to a halt, or be silenced, and then revitalized at a later point” (p12). The precariousness of the Xbank case makes clear the potential for the recycling of value as tissue is circulated and reconfigured as research tissue, new biobanking stock, or incinerated waste. We have extended further our gaze on the silenced and revitalised through an exploration of momentariness as both temporary and perpetual.

From a policy perspective, the Xbank case reminds us that the unanticipated and the unplanned can often form part of the realities of any institutional activity. In a context of significant public investment in biobanks internationally the evident momentariness of both biobanks and bio-objectification raises questions about the sustainability of the field, and of individual biobanks, in the long run. It leads us to ask how many biobanking professionals prepare for facing a situation in which they can no longer afford to pay to keep their holdings, and whether biobanks should develop safeguards to prepare for the large-scale transfer of both tissue and data into new biobanking structures (Tupasela and Stephens 2013). In depth studies by Cadigan et al. (2013) of six US biobanks and a follow-up survey demonstrate a number of instances in which the preparatory practices for closure or transfer of material are limited. When we consider how questions about long-term stability are further heightened by the potential for international repetition of work that may in the long-term render some biobanks as duplicates and unnecessary we recognise the importance of this type of planning.

The challenge is captured in our notion of the momentary, as in both the temporariness of the moment and the recurring, perpetualness, of moment. Momentary as temporariness points to the precariousness of both processes of bio-objectification and the biobanks themselves. The meaning and value of both is premised upon specific modes of socio-technical accomplishment that can potentially reconfigure at any time. Momentary as perpetual points to both the observation that bio-objectification can persist for long periods given secure relationships, and, through Waldby and Mitchell’s analysis of waste, that the recycling of value means bio-objectification can continue beyond becoming waste, through the entanglement in new forms of value. Finally, momentariness as temporary and perpetual also captures a policy tension for biobanks, found in both the Xbank case and those of Cadigan et al. (2013), that biobanks intend to bring perpetual and stable supplies of tissue to research communities, but that as institutions they can experience precariousness themselves that challenges the very meaning and value of the tissue held. For biobanks, and for bio-objectification, momentariness requires a constant attention.
